# Segmenting breast cancerous regions in thermal images using fuzzy active contours

**DOI:** 10.17179/excli2016-273

**Published:** 2016-08-26

**Authors:** Hossein Ghayoumi Zadeh, Javad Haddadnia, Omid Rahmani Seryasat, Sayed Mohammad Mostafavi Isfahani

**Affiliations:** 1 Department of Biomedical Engineering, Hakim Sabzevari University, Sabzevar, Iran; 2 Department of Electronic Engineering, Hakim Sabzevari University, Sabzevar, Iran; 3 Visual Communication Lab, Gwangju Institute of Science and Technology (GIST), Gwangju, South Korea

**Keywords:** breast cancer, thermography, fuzzy active contour

## Abstract

Breast cancer is the main cause of death among young women in developing countries. The human body temperature carries critical medical information related to the overall body status. Abnormal rise in total and regional body temperature is a natural symptom in diagnosing many diseases. Thermal imaging (Thermography) utilizes infrared beams which are fast, non-invasive, and non-contact and the output created images by this technique are flexible and useful to monitor the temperature of the human body. In some clinical studies and biopsy tests, it is necessary for the clinician to know the extent of the cancerous area. In such cases, the thermal image is very useful. In the same line, to detect the cancerous tissue core, thermal imaging is beneficial. This paper presents a fully automated approach to detect the thermal edge and core of the cancerous area in thermography images. In order to evaluate the proposed method, 60 patients with an average age of 44/9 were chosen. These cases were suspected of breast tissue disease. These patients referred to Tehran Imam Khomeini Imaging Center. Clinical examinations such as ultrasound, biopsy, questionnaire, and eventually thermography were done precisely on these individuals. Finally, the proposed model is applied for segmenting the proved abnormal area in thermal images. The proposed model is based on a fuzzy active contour designed by fuzzy logic. The presented method can segment cancerous tissue areas from its borders in thermal images of the breast area. In order to evaluate the proposed algorithm, Hausdorff and mean distance between manual and automatic method were used. Estimation of distance was conducted to accurately separate the thermal core and edge. Hausdorff distance between the proposed and the manual method for thermal core and edge was 0.4719 ± 0.4389, 0.3171 ± 0.1056 mm respectively, and the average distance between the proposed and the manual method for core and thermal edge was 0.0845 ± 0.0619, 0.0710 ± 0.0381 mm respectively. Furthermore, the sensitivity in recognizing the thermal pattern in breast tissue masses is 85 % and its accuracy is 91.98 %.A thermal imaging system has been proposed that is able to recognize abnormal breast tissue masses. This system utilizes fuzzy active contours to extract the abnormal regions automatically.

## Introduction

Breast diseases are among the major issues in women's health today. Early detection of breast cancer is important in reducing mortality rates. Studies show that early detection can lead to an 85 percent chance of survival (Howlader et al., 2015[16]). The chemicals cause difficulties in neural control, and thus lead to regional artery dilatation in the early stages of cancer cells growth (Baba and Câtoi, 2007[3]). This angiogenesis increases the local temperature even a few years earlier than the time of tumor formation (Leung et al., 2009[20]). It seems that deep lesions can cause changes in skin temperature (Kerr, 2004[19]; Leung et al., 2009[20]). Based on the features mentioned above, this concept means that some of the features are adopted in the diagnosis of breast cancer such as geometric size, location, shape, topology and thermal features. Clinicians are interested in thermal imaging because it has the following advantages:

1. Noticeable progressive achievements that have been accomplished in the field of infrared camera technology
2. The creation of standard rules for thermal imaging
3. The accurate calibration of the camera.

Thermography is the measurement of the distributed heat from different parts of the human body. Infrared imaging is a noninvasive method that is used as a diagnostic tool (Arora et al., 2008[2]). Thermogram of a patient provides the distribution of heat in the body. Due to the high metabolic rate and progression of vascular angiogenesis, the cancer cells have a higher temperature than the normal cells around them. Thus, cancer cells in infrared images can be indicated in the form of critical focus. Breast Thermography is a potential technique with useful protocol (Jones, 1998[18]), which has advantages such as being non-invasive, non-radiative, passive, quick, painless, inexpensive, and noncontact camera (Etehad Tavakol and Ng, 2013[9]). Breast thermography is suitable for women of all ages, including pregnant and nursing women and women with dense tissue of breast (Foster, 1998[11]). Therefore, thermography can be useful as a rapid screening test or as a complementary approach. Medical thermography has become a tool for early warning of suspicious breast cancer cases. Discussing the effects of early detection is neither the aim nor the intention of the discussion in this field. The objective is to propose a new model for separating the abnormal breast tissue masses. Techniques and image processing algorithms are the problems of medical applications. These problems may include selecting the best algorithm in order to obtain the best results as well as the optimized image processing algorithms to achieve appropriate responses related to all medical images in this field. Medical thermography could not make progress for a long time because of the dependency on hardware and software and limitations concerning existing. Recently hardware limitations have declined with the improvements that have been made in the field of photo detectors and PCs. Similarly, limitation of software decreased with the progress made in the analysis of algorithms. The above mentioned points have fostered the incremental application of thermal images in medicine (Jones, 1998[18]).

Recently, case studies have been implemented on breast cancer detection in large-scale by thermal imaging, which indicate average sensitivity and specificity of 90 % (Ng, 2009[24]). Parametric analysis is not solved on abnormal areas that are hot and cold points. Thermal imaging is applied in various medical applications such as breast cancer (Qi and Diakides, 2003[28]), pain management (Wang et al., 2003[35]), and diabetes (Cheng et al., 2002[5]). Some methods are also proposed for the analysis of image processing algorithms such as abnormal statistical, thermal asymmetric and descriptive matching methods (Qi et al., 1995[30]). Although the image processing techniques are important in the analysis of medical thermography, many of these methods cannot be considered as sensitive and intricate algorithms to be comprehensively applied in independent detection process through computers.

The purpose of this paper is to provide a diagnostic tool by using thermal based computer technique (Ng, 2009[24]). Previously, various approaches were proposed for the automatic segmentation and detection of thermal images. Methods that have been adopted to separate the abnormal areas in cancerous breast images include: Texture Features and Support Vector Machine (Acharya et al., 2012[1]) and Gabor (Suganthi and Ramakrishnan, 2014[32]). Some major difficulties mentioned in prior studies can be summarized as (Acharya et al., 2012[1]): For breast cancer, the process of detecting and clustering should be done to separate the left and right breast area in thermal image. It is a problem because of the different forms of breast as well as thermal images (small or large, condensation, etc.), and these areas cannot be separated accurately. In this case, the extracted area is not accurate enough for the next step. Some papers (Qi et al., 1995[30], 2012[29]), have used the asymmetry of the two regions and the Moments for separating this area. In the proposed method, this problem has been fixed and there is no need to segment the breast in the images. Identification of the correct boundary and image segmentation is one of the most important issues in machine vision applications. Such as traffic monitoring in urban transport systems (Eng et al., 2008[8]; Huang and Tan, 2010[17]), medical applications (Harandi et al., 2010[14]), video monitoring and target identification in air defense weapons (Chen et al., 2010[4]). Ng et al. (2001[23]) first transformed the breast thermogram images into grayscale, then by implementing gradient filters, found the breast tissue edges. Finally, by dividing the image mesh-wise into 5x5 kernels they investigated the temperature histogram of each region, which was classified by thresholding. Their method is mainly suitable for images which are taken based on standard framework. Some papers such as Ng and Chen (2006[25]), proposed a method for improving the cancerous region, which required investigating the thermal symmetry and the distribution of the skin surface temperature in the right and left breast. Computerized simulations and mathematical analysis are in accordance with empirical methods. Considering this fact, Ng and Sudharsan (2004[22]) modeled the breast tumor region. In their method, a bio-heat model of female breast region is proposed. Although the proposed model includes coefficients regarding the thickness and contents of the tissue and some other factors, but several combinational parameters are missing in the equations. Other models (Sudharsan and Sudharsan, 2001[31]) have also used a numerical scheme to achieve a real model. In other similar articles (Ng and Sudharsan, 2001[23]) three dimensional thermogram models of breast cancer have been simulated. Etehad Tavakol and co-authors (2013[10]) evaluated the diagnostic classification of thermal images into malignant, benign and normal classes using the effectiveness of bi-spectral invariant features, and proposing a phase-only variant of these features. Their method involved a technique for separating edges using the c-means fuzzy clustering method. Although the proposed method has an accuracy of 95 % for detecting malignant cases, but the investigated number of images were 9 out of 32, that according to the formula for calculating sample size, the number of investigated samples were relatively small. In another article from the same author (Golestani et al., 2014[13]), the investigation and classification of cancerous regions, especially of fibrocystic images applied k-means, c-means then the level set method was discussed. Unfortunately, their proposed method has been conducted on limited samples. Summing up the mentioned papers, it is noteworthy that the proposed methods relate only to the specific region of the breast thermal images and considers only a limited population. 

## Objectives

This paper aims to initially detect the abnormal masses of breast tissue and then segment these regions using active fuzzy contours. Another significant point unlike previous papers is that a standard approach has been introduced considering all the image artifacts such as noise, disturbance, etc. For segmenting the cancerous area in an effective way, two important sections of the proposed technique include the separation of the thermal edge from the core of abnormal areas.

The novelty of our technique can be pointed out as below:

Collecting complete thermal images dataset of patients who have the symptoms of benign breast cancer for the first time in Iran in such a big scale.

Proposing a new method with the ability to segment the abnormal regions into the core and the edge.

Enhancing the active contour using multiplication weights, Lyapunov exponents and etc. Consequently, the active contour can result in a closed region. Generally, splitting the thermal image to its RGB values and gray-level does not result in a final closed region because of the nature of this kind of imaging, and the core boundary could not be recognized.

Creating the appropriate fuzzy rules regarding the personal technical experience in thermal imaging and comparing state of art methods.

## Patients and Methods

It is noteworthy that the mentioned 60 cases investigated in this paper are images which the authors have collected through a clinical and systematic process. The following steps were implemented in the present study to collect the images: First, 60 patients referred to Imam Khomeini Hospital Complex (Tehran) which were suspected having breast tissue diseases - considering the type of their disease - were experimented by mammogram, ultrasound and biopsy tests. Before conducting thermal imaging, different parameters which would have unwanted effects in the thermal imaging results such as the imaging condition, room temperature, patient's convenience, conducting thermal imaging before ultrasound imaging and etc. were carefully considered. The patients were asked to fill up a questionnaire containing their age, weight, consumed medications, menstruation cycle, previous cancer history and other useful information regarding breast cancer. After determining these initial requirements, the thermography image acquisition was performed. The patient was asked to undress the upper body completely. Then a moment was given for the patient's body to be in thermal equilibrium conditions. Afterwards, the patient was asked to hold up her hands and the operator took the thermal image of the patient's breast. The model of the used camera is Infrec R500.

In this study, after acquiring and saving the patient's history, the results of clinical examination, ultrasound stereotypes, biopsy and thermography images were evaluated independently by the relevant experts. And after saving the results, considering a specific clinical sign, the abilities of thermography imaging was compared in being able to detect healthy subjects from patients having cancer. Overall, 60 patients were studied with an average age of 44.9 years, with the youngest age of 21 years and oldest of 73 years old. The standard deviation was 11.56 based on our calculations were the lower and upper confidence bounds are -0.2011 and 0.2011 respectively. 

**Figure 1:**
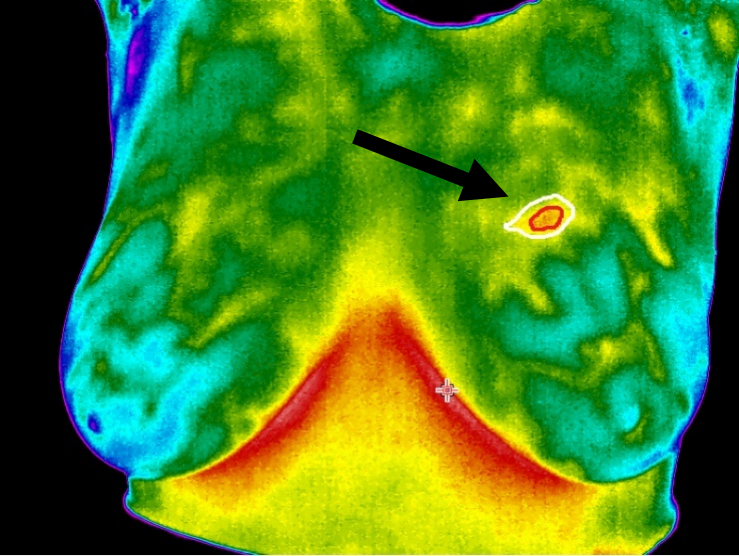
The white and red areas pointed by the arrow correspond to the thermal edge and core of the cancerous tissue mass respectively

Initially a method is proposed based on the active contour for segmenting thermal edges, and then the original model is offered to isolate the core of the abnormal area. At this point, weighted coefficients of active contour act as a fuzzy logic model. In this paper, we focus on parametric active contour models, and then we propose two novel energy functions for detecting the thermal edges and cores in abnormal areas. The original thermal image includes temperature pixels which can be converted to RGB format. The proposed method has been applied in both models. A thermal image of the breast area can be seen in Figure 1(Fig. 1). The area related to the abnormal core has been shown at the tip of the arrow in red color and the thermal edge has been shown in white.

### Detection of the abnormal thermal core 

Although many methods have been proposed for noise and ambiguity reduction in thermal images, it is still a major concern. Based on the aforementioned points we represent a novel model to detect the thermal core. First of all, we elaborate on our feature extraction method of autocorrelation function for 1-D signals. We attribute this concept to 2-D signals and put forward a novel feature image. Subsequently, this new feature is combined into the energy function of parametric active contour, and we propose a new energy function for thermal core boundary detection and the abnormal thermal edge.

We can delineate the autocorrelation of a stationary discrete process f(m) at lag k as (Peebles et al., 2001[27]):


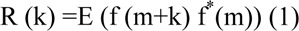


The levels of f and f* for a real function are similar. f* is the complex conjugate related to f and E is the expected value operator. In such a way that the function for an ergodic real signal can be defined as the following equation:


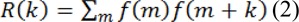


These types of actions represent the resemblance among signal f(m) and a shifted version of itself. The higher value of autocorrelation indicates that the main signal and the shifted signals are highly interrelated and its less value indicates that it bears slight similarity. In order to detect the period of repetitious incidents, autocorrelation function can be used, because it gives highly applicative data about periodic events. But it isn't suitable, since it cannot be applied as a feature for each point of the signal. The short-term autocorrelation function can be applied as a perfect feature, in which the signal is windowed to overlapped or non-overlapped small parts. The equation of short-term autocorrelation related to a real signal is as follows (Deller Jr et al., 1993[6]):


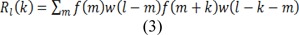


**Figure 2:**
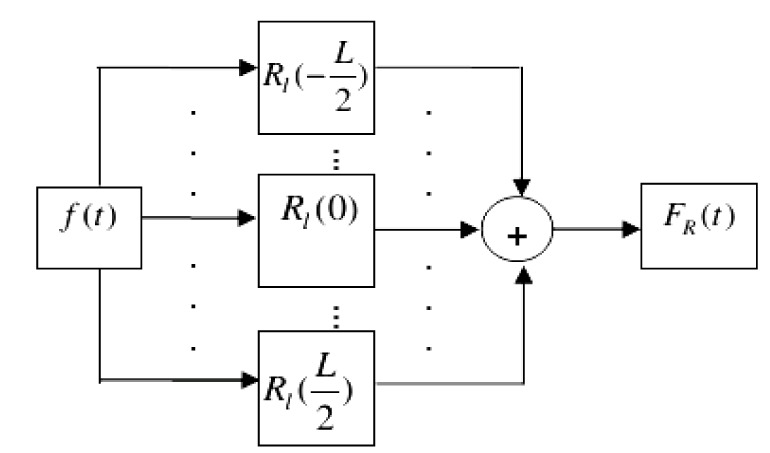
ASTA computation flowchart for a small window around index t of the signal f

Where w(l) is recognized as the small window related to length L. Also, the Short-term autocorrelation aboard R_l_(-L/2) to R_l_(L/2) around each index t associated with the signal f(t) is computed. Next, the total value of the gained short-term autocorrelation is calculated. In the next stage, it is supply in position t. The respective operation has been shown in Figure 2(Fig. 2). The outcome is an updated function F_R _which is created by the summation of short-term autocorrelation. Accumulative Short-Term Autocorrelation (ASTA) is the name that we give to this new function.

Based on the signals defined at lag k_1_ and vertical lag k_2_ for the image I, there is a possibility to extend ASTA to 2-D image signals. Our proposed method in this stage uses coefficients p_t1_, p_t2_ influenced by temperature pixels, defined as following:


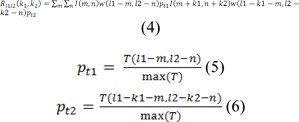


Where w(l_1_,l_2_) and (pt1, pt2) are a 2-D small sliding windows with size of L˟L, and the temperature of pixels in the image respectively, with the Size of small sliding window which is L×L. In our suggested method, initially we calculated 2D-STA quantities for pixels (m,n) linked with image I, that it is in a small window around the pixel from R_l1l2_(-L/2, -L/2) to R_l1l2_(L/2, L/2). Then, the aggregate of the 2D-STA amounts should be normalized, it should be noted that the normalized range is between 0 to 255. Subsequently, it is supplied in the location index (m,n). In the consecutive procedure, we proposed a new feature named FRN, with exactly the same size as the main image I and the values equal to the normalized aggregate of 2D-STAs. We conceptualize this new feature image as normalized accumulative short-time autocorrelation (NASTA), which is suitable for abnormal thermal edge detection in images. Diagnosing the abnormal thermal core is a very complex procedure. NASTA can detect the boundary linked with the thermal core. It is noteworthy that the NASTA values can provide a novel NASTA-based pressure active contour or fuzzy active contour model. In our proposed method, a pressure active contour model was selected, because it is less sensitive to weak or blurred edges. In addition it is convergent in case of concave boundaries. The energy function for border coupled with thermal edge detection is as below. Overall, we set it based on NASTA and inspired by the pressure active contour model (Vard et al., 2012[33][34]):


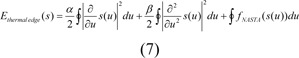


The second terms in the equation are in connection with internal energy and control the contour shape and S(u) is a parametric curve in Cartesian coordinates. It should be taken into account that it is the first-order and second-order derivative control contour stretching and bending respectively. In order to control the contour's tension and rigidity, from α and β, the weighted coefficients are adopted, respectively. α and β are calculated based on fuzzy logic that will be discussed below. The third concept used widely in the equation is the image energy delineated as NASTA pressure energy. f_NASTA _is the NASTA pressure force in the energy equation which can be illustrated as follows:


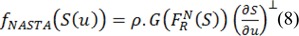


In this equation, since the NASTA pressure force is perpendicularly utilized in the tangent of the contour, ⊥mark is used. Also q is the weighting coefficient. We can represent the function of G as follows:


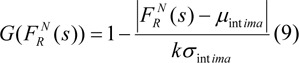


Where k is a positive parameter which it set by the operator. µ_intima_ and σ_intima _include the mean and standard deviation the NASTA levels of the thermal edge area, respectively. Also


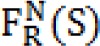


is the level of NASTA corresponding related to the contour position S. As another important factor, the energy of nominalization of E_thermal edge_(S) which is widely linked to S is implemented by exploiting the iterative gradient descent method, and is also revitalized for each point of contour using the following equation:


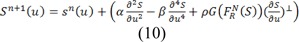


Where S^n^(u) is taken into account the positions of the contour.

### Detection of the abnormal thermal edge 

We applied an energy function for the active contour model, so that we can segment the border between thermal core and edge. The outlined Energy function is depicted as:


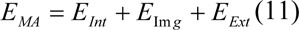


Where E_Int _is the internal energy which is considered as:


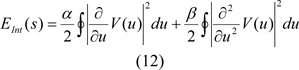


Where V(u) is a parametric curve and ∮ Closed curve line integrals. Taking into account edge information as well as texture features, the image energy is outlined as:


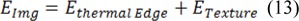


We can calculate edge energy based on the following:


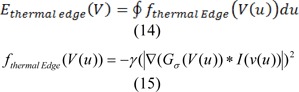


Where * is the convolution operators, γ is the positive parameter and I(V(u)) is the image intensity at contour point V(u), also Gσ is a 2-D Gaussian kernel with standard deviation, and σ.∇ denotes the gradient. In order to reduce the impact of noise, Gaussian filtering is used (Dijkstra et al., 1999[7]). Initially the texture features should be extracted from the input image, so as to be able to compute texture energy. In this stage, texture features are extracted with respect to the semi-local image information, together with the metric tensor, which is put forward in (Zhang et al., (2010[36]). This description obtained from texture indicates lower level of sensitivity to noise; therefore it can be used as features that it applied for IVUS images. First of all, the square window around the pixel (x,y) of the image I with size of w _˟_ w should be defined as:


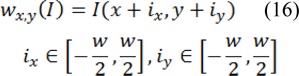


In the next step, we apply the agency related to textures in the Beltrami framework pursuant to Houhou et al. (2009[15]) as follows:


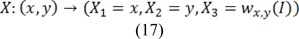


This mapping can offer a position in space, in coordination with local data and window values around the current pixel as semi-local thermal image data. Afterward the related metric tensor linked with [15] is formulated as follows:


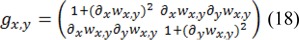


In the end, we can elucidate texture descriptor as:


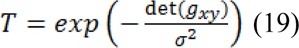


Whether


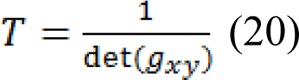


Where det (g_xy_) is the determinant of g_xy_ and σ > 0 is a scale parameter. In the next stage, we merge the pressure force with obtained texture features, so the texture pressure force descriptor was defined as:


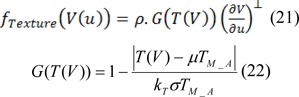


Where µ_TM_A _and σ_TM_A _are the mean and the standard deviation pertinent to texture feature levels. k_T_ is taken as positive parameter which applies by using of operator, and ultimately T(V) is the value of texture feature. In the subsequent stage, texture energy is calculated as follows:


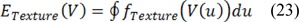


In some cases segmenting thermal edge of abnormal area is complex procedure, and it would be more difficult if there were shadows and side branches. In order to solve these problems, the detected contour for border of thermal edge is put to work as an initial contour. Accordingly, an external energy is defined as follows:


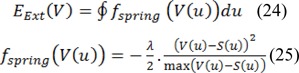


λ and S(u) are a constant quantity as well as the parametric active contour. It is noteworthy, that S(u) is the detected border of thermal edge in the previous stage. f_Spring_ is indicated as spring force. As a result, we set the update equation for each point with regard to the contour which is performed by using an iterative gradient descent method as follows:


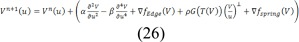


Where V^n+1^(u) is the position related to the contour point V in iterations n+1.

### The process of growing of the fuzzified contour 

**Figure 3:**
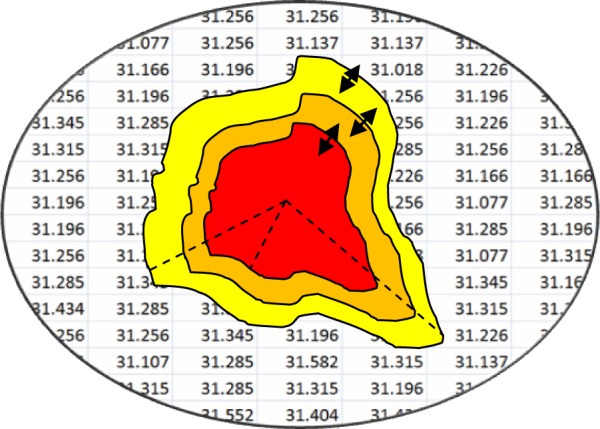
Temperature distribution of tissue in the abnormal area which will apply fuzzy models on active contour

As previously pointed out, α and ß are weighted coefficients which control the sensitivity of the contour to the stretch and curvature. Considering the fact that the contour keeps growing in the absence of an enclosed boundary, the proposed method can be used to stop the growth from one thermal zone to another. The feasible idea in this step is that a fuzzy logic model should be implanted with respect to the experience obtained in the collection of dataset. In other words, the fuzzy model is computed and the relevant zone is identified each time when the contour grows. So it can be an appropriate factor to stop the growth. In addition to this important efficiency, the same model should be applied to calculate the weighted coefficients α and ß and the impacts of which will be shown properly. Therefore, the values of weights should be updated in each iteration. The initial values of α and ß are within the range of zero to one at the beginning of the contour. This procedure continues until the threshold is reached after a while. Put another way, the values of weights should not be any different, or the contour should reach a zone of thermal pixels. Figure 3(Fig. 3) indicates the distribution of tissue temperature in an abnormal area applying the fuzzified model of the active contour. 

**Figure 4:**
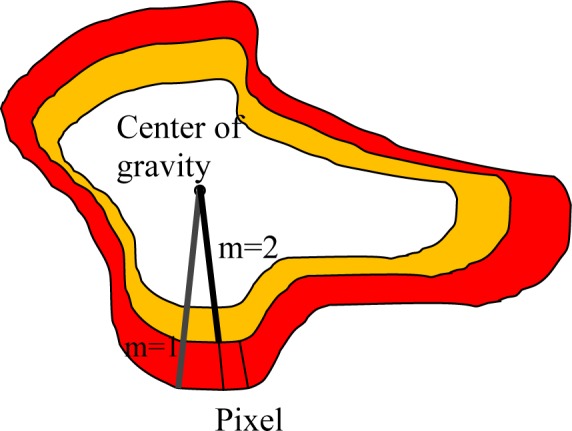
Simulating the cancerous area based on the thermal core and thermal edge

To express the problem accurately, the area related to cancerous breast is simulated as in Figure 4(Fig. 4). The thermal core is separated from the thermal edge.

### Temperature changes in the thermal edge

One of the inputs provided in the collection of dataset is the temperature change in thermal layers located in the distance to the unnatural center and core. The temperature means of thermal layers decrease in value in zones which are farther from the center of the core. Change in the temperature of layers of thermal edges will be one of the important factors in the active contour of thermal images. Table 1(Tab. 1) indicates the range of temperature changes obtained by analyzing the thermal images of breast.

**Figure 5:**
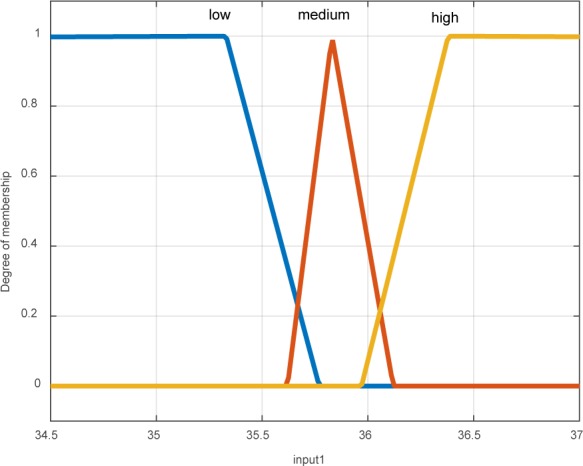
Membership function of temperature changes

According to Figure 4(Fig. 4), the temperature mean of each layer in the extension of contour is calculated to obtain the membership coefficients in the fuzzy system pertaining to temperature fluctuations in these layers. The means were divided into three ranges including high, medium, and low temperatures (Figure 5(Fig. 5)).

### The rate of the growth in the examined layers

The second input discussed in the fuzzy control, was the area of zones pertaining to the layers investigated in the growth of contour. As previously mentioned, the thermal zones, observed in the area of breast tissue masses, were comprised of enclosed thermal layers (resembling circular zones) growing in size in the zones farther from the center of the core. However, the considerable point is that the growth area of each zone is in relation to the area of the previous zone. This rate should not be greater than a specific value because the contour exceeds its zone in the relevant layer, a fact which may prevent the identification of edges on the initial image. According to the previous investigations in the zones pertaining to the core, the contour grows almost uniformly; therefore, the changes will be slight. However, the growth rate increases in the areas farther from the core due to morphological and structural changes.


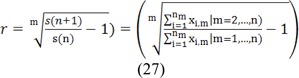


**Figure 6:**
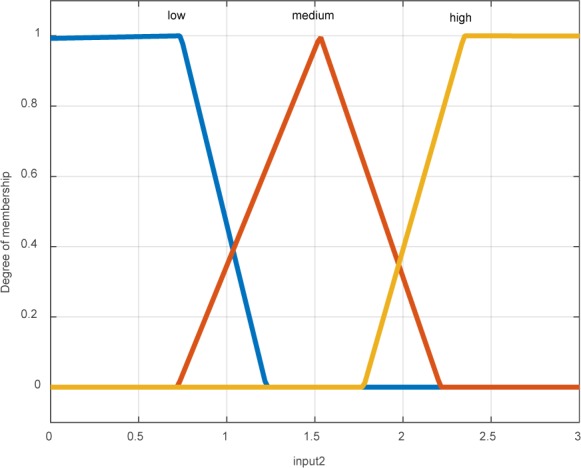
Membership function related to changes of the area in the surrounded layers

The value of x is “1” when the desired pixel is located in the studied area. The value of m is equal to the implementation of m growths of the active contour in one thermal layer, and r is the rate of area growth in the implementation of contour. In better words, the above relation indicates the growth rate of changes in area in the step n+1 compared to the step n. Figure 6(Fig. 6) shows the diagram pertaining to the membership function of the input rate of area growth.

### Behavior of abnormal area 

The third factor was the behaviors of unnatural zones (being chaotic or not). According to the morphological structure obtained in the collected database, it can be used to identify the uniform behavior of an unnatural thermal core. Put another way, the behaviors will be more chaotic in the areas farther from the core. The proposed method enables us to diagnose the structure of the isolated tissue. The detection is performed using Lyapunov exponent test model. By this test it is possible to distinguish accidental from non-accidental time series without statistical distribution. According to this test, we can express the similarity of data in a time series, so that period of effect data appears to predict the future. This test is described as follows. Suppose we have N observation of a chaotic time series available as follows:


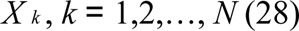


In this case, to obtain the Lyapunov exponent, divided M vectors is initially formed from these time series as follows:


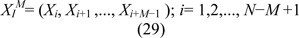


Then among these vectors, all pairs of vectors proved in the following equation, are determined:


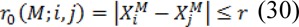


Where r is a small positive number and ‖.‖ is the 2-norm, which indicates the distance in Euclidean space. Value of r is considered “1”. Now with transfer time to the n-step ahead, this distance is computed again, thus:


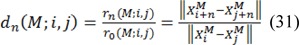


If these vectors are far apart we result that d_n_ is the larger than one, otherwise, smaller than 1. Thus, the largest Lyapunov exponent is calculated as follows:


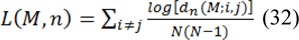


**Figure 7:**
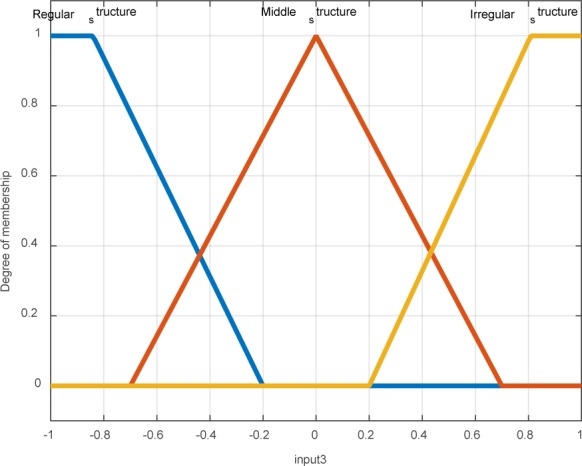
Membership function related to the behavior of an abnormal area

The positive value of L indicates that time series is chaotic and predicted divergent. A negative value of L represents a definitive nature for time series and long-term predictability. So if L accepts positive values close to zero, the system is weakly chaotic. And medium-term prediction is possible, but predicted long-term is impossible. Up to now, the following steps are done using original image. First, the distance of the center related to the segmented area from its edge is calculated as time series and distance values are written in the same way. Then Lyapunov exponent test is implemented. The membership function related to the behavior of an abnormal area is shown in Figure 7(Fig. 7).

**Figure 8:**
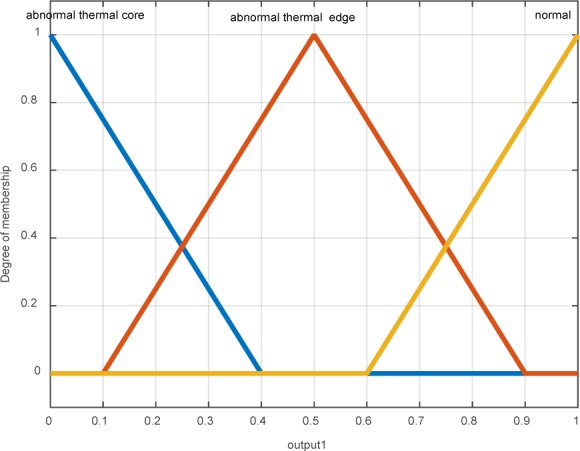
Output membership functions which contain three abnormal thermal core, abnormal thermal edge and normal area (outside the area)

The desired output is discussed in three main states: 1- Normal: in this case, temperature changes are low and outside the unnatural range and relevant thermal layers. 2- Thermal edge: it is the zone, separated by the contour, enclosing the thermal edge of unnatural zone. 3- The unnatural central core: it is the zone, separated by the contour, including the core of unnatural thermal edge (Figure 8(Fig. 8)).

A fuzzy rule data base is composed of a set of fuzzy rules with the if-then format. According to the structure knowledge, the mapping behavior of the output (µ = α and ß) can be described by means of eight fuzzy rules, shown in Table 2(Tab. 2). The input variables are temperature, area changes and behavior of abnormal area that there are totally 25 fuzzy rules. 

The output has 4 modes:

1. Normal: In this case, the temperature changes are low
2. Suspicious: The separated area is suspicious related to abnormal thermal core or core edges
3. Abnormal
4. Highly Abnormal.

The last two outputs are very important in the edge of active contour. 

**Figure 9:**
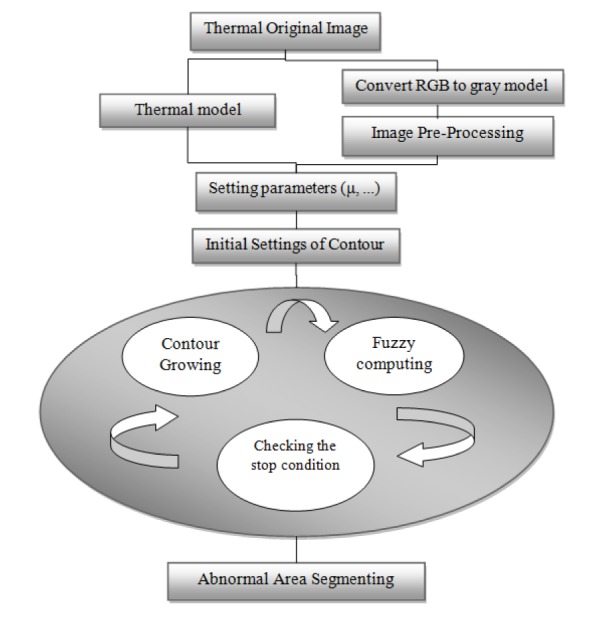
Overall flowchart of the proposed method's stages

Figure 9(Fig. 9) indicates the flowchart pertaining to the algorithm of the proposed method. In this algorithm, the initial configurations of contour are first set with respect to the zone which should be separated. In the initial steps, the contour starts growing towards the boundary. After a few steps of growth, the fuzzy cases are calculated: 1. temperature changes in the thermal edge, 2. the rate of the growth in the examined layers and 3. behavior of abnormal area. If the relevant boundary is reached, the contour stops growing. Otherwise, the process of fuzzy computation is simultaneously iterated at each step of the growth of contour. It is worth mentioning that the termination condition of contour can be discussed in two ways. In the order of priority, we have: 1- the transfer and replacement of the boundary with the use of the fuzzy model, and 2- termination under the condition of reaching energy balance in the main relationship of active contour.

Based on the proposed algorithm's flow diagram, the taken thermal image is transferred from its RGB values to a gray-scale image. The next step is image pre-processing which is done to edge RGB image using “canny edging”. Next, the initial parameters of the contours are set. Values of α and ß are considered as 1 in the Start of working. The value of *ρ* is considered 1 due to the good results that are obtained. In the first stage, the starting point of the contour is delivered. This means that firstly, we define closed border on the abnormal area (defining closed border is essential characteristics of Active Contour). By starting the contours functionality, the calculations regarding the multiplication weights and the Lyapunov exponents which are related to the fuzzy contour parameters are processed and turn into limits or conditions for the active contour. The contour continues its progress until it reaches its energy balance which is the closed boundary of the thermal edge. At this stage the abnormal region has been detected.

In order to analyze the obtained results, we compared the segmented outputs from the proposed method with manually traced results (Papadogiorgaki et al., 2008[26]; Zhu et al., 2011[37]). Suppose A = {a_1,_…,a_p_} and M = {m_1_, ,. . . , m_q_} are the automatically detected and manually traced contours respectively. The distance between the point a_i _ϵ A and the contour M is defined as:


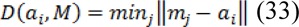


According to this, two distance measures are calculated as follows:

1. Mean Distance (MD):


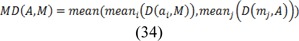


2. Hausdorff Distance (HD):


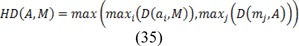


The Hausdorff distance is the maximum distance of point to point contour variations in the same venue. Mean distance is the average distance of point to point contour variations in the image. Dataset 1 (Ghayoumi Zadeh and Baghdadi, 2015[12]) is used for testing the proposed method. To evaluate the proposed method, some statistical analyses on efficiency metrics computed from the results of our model and manual tracing has been applied. To validate the effectiveness the proposed algorithms, HD, AD were calculated as the performance measures between the manual tracing and the outcomes reached by the proposed fuzzy active contour for 60 cases. Since our initial aim of the proposed method is to extract the abnormal regions therefore, it was required to choose the subject patients of thermography from those who already had abnormal masses in the breast tissue.

## Results

Since the investigating subject were breast tissue masses all the samples were affected by cancer. The patients 'pathological results' included 39 cases of hypoechoic mass, 14 cases of cystic mass, and the remaining 7 patients had specific symptoms, 3 cases had heterogeneous, fibroadenoma, intraductal mass and 2 cases had fibroglandular and spiculated breast mass and finally 2 cases had ISO echo with cystic and calcified mass. The analyzed images can be classified based on the Thermo-biological scores into five main groups: TH1 - Normal image without vascularity (blood vessels), TH2 - Normal image with vascularity, TH3- Equivocal (questionable, but not abnormal), TH4 - Abnormal TH5 - Very abnormal. Table 3(Tab. 3) presents the results of classifying thermal images on 60 patients based on their Thermo-biological scores. It is observed that asymmetry and thermal class are directly related to each other.

**Figure 10:**
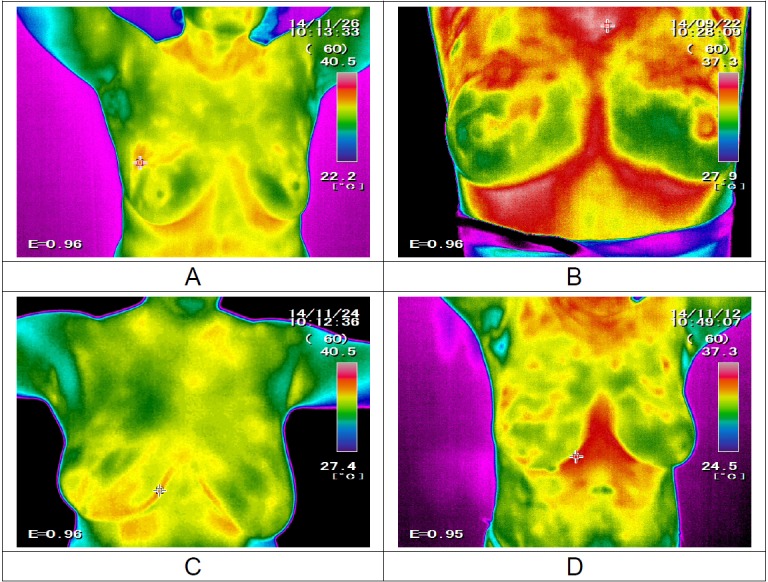
The thermal images of: A - hypoechoic, B - cystic, C - heterogeneous and D - fibro-glandular masses

Figure 10(Fig. 10) presents the thermal images of hypoechoic, cystic, heterogeneous and fibroglandular mass.

We will reach some results if initial diagnosis using thermography is gained by the symmetry technique (Qi et al., 2012[29]). The sensitivity of IR imaging to tumor type, age and size is shown in Table 4(Tab. 4). Due to these results, it is clear that by increasing age, cancer detection will become more difficult.

**Figure 11:**
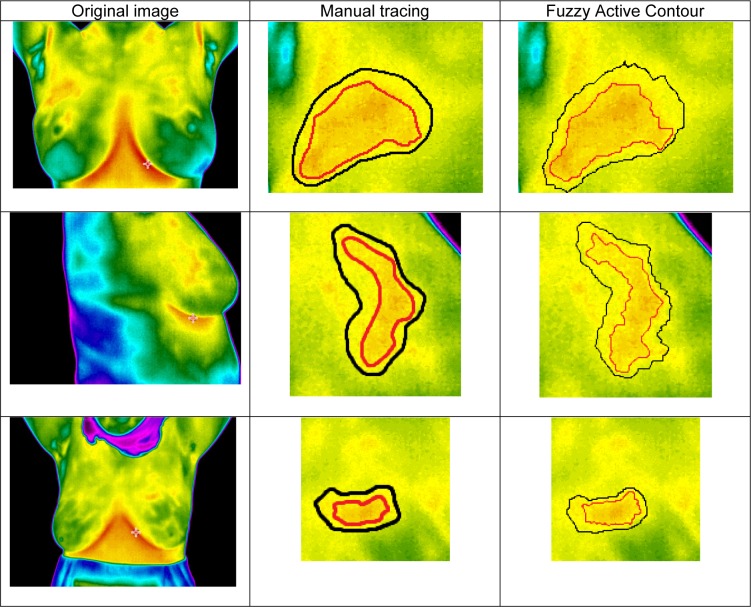
Comparison of the segmented area using Manual Tracing and using Fuzzy Active Contour. Black and red lines are related to the thermal edge and core respectively

In Figure 11(Fig. 11), the output results are shown. It should be noted that only the cancerous area is examined.

As Figure 8(Fig. 8) expresses, the output result of the proposed method using fuzzy active contours in extracting abnormal tissue regions of the thermal breast images are very similar to the region extraction of manual tracing by the expert. Statistically comparing, based on Table 5(Tab. 5) and 6(Tab. 6), the outcomes of the methods are rather discrepant with the manual tracing results.

In Table 5(Tab. 5) and 6(Tab. 6) are comparing the Mean Distance (MD) and Hausdorff Distances (HD) between Expert1 (E1), Expert2 (E2) and the proposed Fuzzy Active Contour method (FAC).

**Figure 12:**
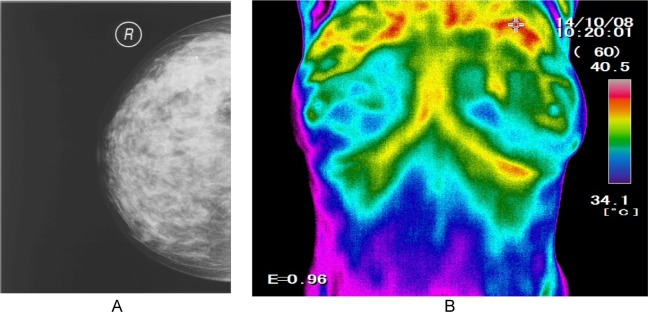
The mammogram (A) and thermogram image (B) of a mass on the upper part of the right breast

All the investigated thermography images were tested by their mammogram and ultrasound relevant pairs. A sample thermogram image with its relevant mammogram image is presented in Figure 12(Fig. 12) for reference. The medical experts' diagnosis in this special case when considering the craniocaudal and lateral views of the mammogram images was: “A dense mass without micro calcification was seen in the upper part of the right breast”. Which is identical to the thermograph which the designed system has captured.

The Statistical analysis and comparison regarding the thermal symmetry has been brought in Table 7(Tab. 7). Furthermore, the comparison of Hausdorff distance between the manual method and the proposed method, and the Hausdorff distance between the manual method and conventional algorithms are represented in Table 8(Tab. 8). Once again, our initial aim of the proposed method is to extract the abnormal regions therefore, it was required to choose the subject patients of thermography from those who already had abnormal masses in the breast tissue. If not analyzing terms such as specificity and NPV (negative predictive values) in this paper were meaningless. Sensitivity refers to the test's ability to correctly detect patients who do have the condition and specificity relates to the test's ability to correctly detect patients without a condition, which are calculated according to the following equation:


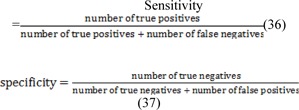


## Discussion

In this study, we selected 60 thermography images with various modalities acquired from different patients for validating our algorithms. These pictures are different forms of cancer tissue masses including cystic masses, etc. We tested the proposed method for segmenting thermal edge and core on patients in order to validate this method. The results show the accuracy and effectiveness of the proposed method for segmenting the thermal edge and core in images. However, it is possible that after further experiments on more images, the accuracy of the proposed method decreases.

In previous research, many methods have been proposed to form a mathematical model for pattern classification. In this paper, we presented a method of completely automatic segmentation that can diagnose the breast cancer core in the breast thermal images. The proposed method is presented for segmenting abnormal thermal core and edge for the first time, so it cannot be compared with previous works. It should be noted that the previous methods can be compared only on thermal core.

## Conclusion

The abnormal temperature of body is the natural indicator of the disease. Thermal imaging (Thermography) utilizes infrared beams which are fast, non-invasive, and non-contact and the output images by this technique are flexible and useful to monitor the temperature of the human body. In recent decades, extensive research has been conducted to increase the use of thermal cameras and to obtain the close relationship between thermal physiology and skin temperature. Generally, the most original and difficult task in image analysis includes both the segmentation and separation stages. With effective segmentation, the desired component is separated. The superiority of one method over another in segmentation depends on the special specifications of issue that is to be checked. In some clinical trials, and biopsy tests, it is necessary that physicians and scientists know the cancerous enclosed area in the breast tissue.

In these cases, the thermal edge detection is of special significance. To put other way, by a detailed assessment of breast cancer, delectation of cancerous core or abnormal area in the thermal image will be of unique quality. In this work, the intelligent approach is presented for both the thermal core and edge detection in abnormal area. Two new energy functions are presented for parametric active contour in order to automatically detect the edges in the areas of abnormal thermal core and edge. In this paper, we presented a new model of fuzzy active contour along with the programming techniques in contour to segment abnormal areas of the breast cancer. In this regard, the important point to stress is the use of heat distribution for increasing accuracy in segmented areas, as well as creating the closed border for contour model. Finally, the segmented results of the presented method are compared with manual method by the help of the Hausdorff and average distance. Consequently, there is limited difference in the obtained results, implying that the proposed method is appropriate. In addition, the statistical analysis of the sensitivity and accuracy of the proposed model were assessed and results were satisfactory.

## Figures and Tables

**Table 1:**
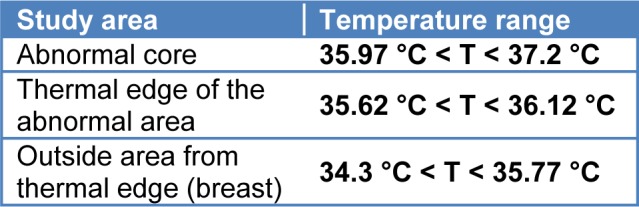
The range of temperature variation in the areas examined breast tissue

**Table 2:**
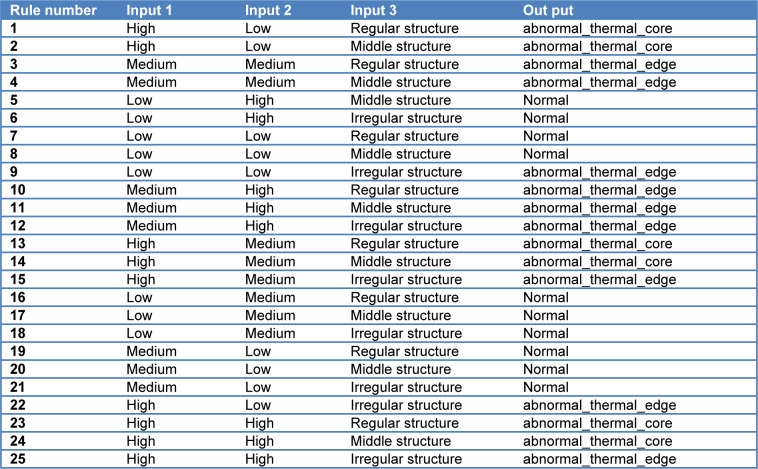
The fuzzy rules based on input and output

**Table 3:**
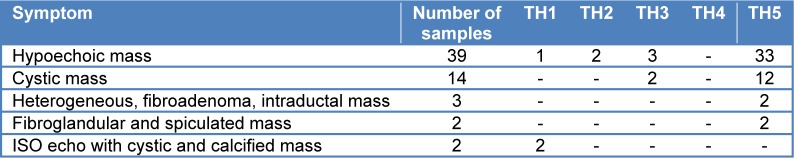
Classifying the subject samples based on symptom types and their standard Thermo-biological scores

**Table 4:**
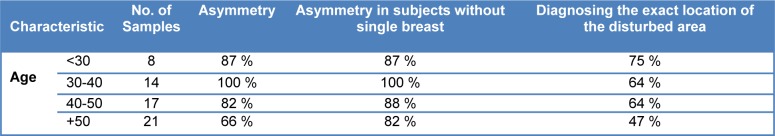
The sensitivity of the detection of abnormal are as using asymmetry techniques with respect to the age of patients in the thermal images

**Table 5:**
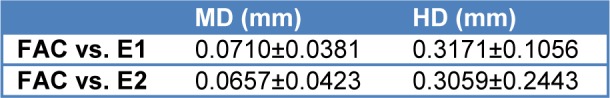
Calculation of Hausdorff and average distance between the manual and proposed method in area of abnormal thermal edge related to test run twice

**Table 6:**
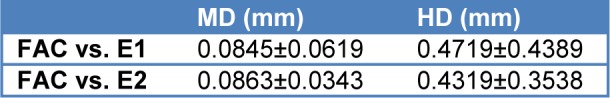
Calculation of Hausdorff and average distance between the manual and proposed method in area of abnormal thermal core related to test run twice

**Table 7:**
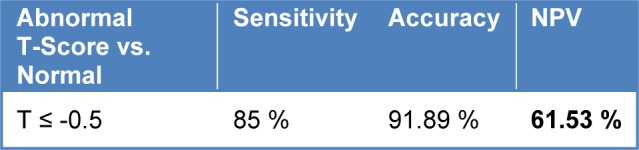
Statistical analysis of the thermal non symmetry

**Table 8:**
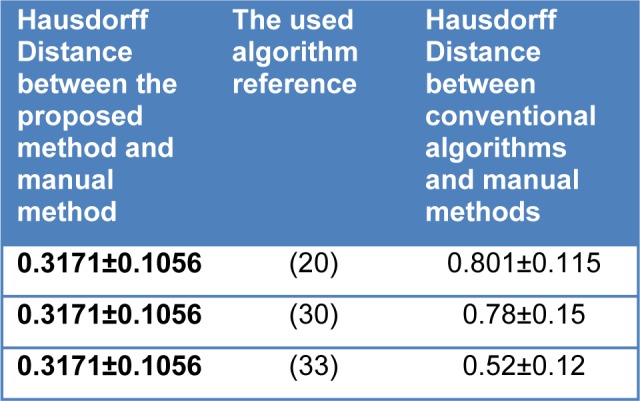
Comparing different conventional algorithms of active contour with the proposed method (given in millimeters)
